# Refugees Connecting with a New Country through Community Food Gardening

**DOI:** 10.3390/ijerph110909202

**Published:** 2014-09-05

**Authors:** Neil Harris, Fiona Rowe Minniss, Shawn Somerset

**Affiliations:** 1 Population and Social Health Research Program, Griffith Health Institute, Gold Coast Campus, Griffith University, Gold Coast, Queensland 4222, Australia; 2 School of Medicine, Gold Coast Campus, Griffith University, Gold Coast, Queensland 4222, Australia; E-Mail: f.roweminniss@griffith.edu.au; 3 School of Allied Health, Faculty of Health Sciences, Australian Catholic University, Brisbane, Queensland 4014, Australia; E-Mail: shawn.somerset@acu.edu.au

**Keywords:** refugee health, nutrition transition, community food garden, social connectedness

## Abstract

Refugees are a particularly vulnerable population who undergo nutrition transition as a result of forced migration. This paper explores how involvement in a community food garden supports African humanitarian migrant connectedness with their new country. A cross-sectional study of a purposive sample of African refugees participating in a campus-based community food garden was conducted. Semi-structured interviews were undertaken with twelve African humanitarian migrants who tended established garden plots within the garden. Interview data were thematically analysed revealing three factors which participants identified as important benefits in relation to community garden participation: land tenure, reconnecting with agri-culture, and community belonging. Community food gardens offer a tangible means for African refugees, and other vulnerable or marginalised populations, to build community and community connections. This is significant given the increasing recognition of the importance of social connectedness for wellbeing.

## 1. Introduction

Populations that migrate from one country to another, in particular from a less industrialised to a highly industrialised country, undergo a consequent nutrition transition associated with changes in their physical and social environments, diet, physical activity, and access to healthcare [1,2]. Evidence suggests that on arrival migrants from developing countries typically have better overall health than the non-migrant population, often referred to as the “healthy migrant” paradox. It is paradoxical as, over time, migrant health status is prone to decline [3,4] as this population faces settlement difficulties associated with access to appropriate healthcare, language difficulties, financial difficulties as a result of unemployment, and cultural differences [5]. Influenced by such factors as low income, media and accessibility, food choices are a common cause of poor health among migrants, as they shift from a traditional “wholesome” diet, to a more refined, high caloric “western” diet [1]. Refugees are a particularly vulnerable category of such populations, whether moving from their traditional homelands to temporary refugee camps or settling in an industrialised host country.

The health of humanitarian migrants in Australia and other industrialised countries is relatively poor compared to sedentee populations [6,7,8]. African refugees comprise more than 30% of the Humanitarian Program annual intake in Australia [9]. African humanitarian refugees represent a relatively recent and growing sub-population in Australia with most migrating and settling in the past 15 years [10] as a result of conflicts arising from decolonisation and government instability [11]. Of the migrant sub-populations of Australia, the Sub-Saharan African community has the highest proportion of children aged 0–14 years (12%) [12]. Most of the Sub-Saharan African refugee population identify as Christian, and are women with young children and families, with limited English and educational attainment [11]. Approximately 40% of all Humanitarian Migrants living in Queensland reside in homes with between 5–7 people [13].

Many African humanitarian migrants have experienced serious human rights violations as a result of war and organised violence, as well as extended stays in refugee camps. As a result of their cultural transition and traumatic experiences, many African refugees suffer from significant social displacement, as well as psychological and physical health problems [9,14]. Compounding their social displacement and disconnection, many refugees come from farming backgrounds, and their forced migration to a “landless” urban environment presents additional challenges [15].

Renzaho and colleagues [15] noted a dual burden of overweight/obesity and under-nutrition in a group of refugee families from sub-Saharan Africa. Other studies, for example on Liberian, Somali and Vietnamese refugees at different locations, have reported on the difficulties these groups have in establishing healthy food habits upon settlement [16,17,18]. The food consumption habits adopted by such people upon settlement in Australia and other highly industrialised nations [19] are likely to intensify the consequences of their already compromised health status. The development of appropriate lifestyle and health interventions for refugee populations settling in Australia is a major challenge since access to and engagement with these populations is complicated by a range of cultural and social barriers [20]. Furthermore, while this population establishes early social connections within their own ethnic communities, as a disparate collection of vulnerable groups they often have limited meaningful social connectedness beyond these communities [9].

In the past decade community food gardens have increasingly been implemented as a localised urban intervention to improve access to low cost nutritious food, physical activity and community networks within urban populations in industrialised country settings [21,22,23,24]. Community food gardens contribute to the various needs of communities and are used not only for growing of nutritious foods, but also for such things as leisure, crime prevention, healing therapies, and ecological restoration [25]. The growing body of research and practice around community gardens highlights their roles in re-building localised urban food systems, contributing to food security and as part of the social mobilisation required for sustainable development [25,26,27]. Of particular note for the current research is the tendency for community gardens to include marginalised populations in social, collective endeavour where they can acquire skills, access nutritious and culturally relevant food and enjoy the physical and psychosocial benefits of tilling the earth [21,22,23,28,29].

In the context of the demonstrated effectiveness of community food gardening to promote community connectedness, this study explored how involvement in a community food garden supports African humanitarian migrant connectedness with their new country. Given the exploratory nature of the research, a qualitative approach was adopted with the study framed as a single critical case study [30,31] conducted on the community food garden sited on the grounds of a University campus in the City of Logan, Australia. A case study approach was adopted as a recognised strategy to develop understanding and explanations within social science research [32].

## 2. Methods

### 2.1. Study Setting and Population

**Figure 1:**
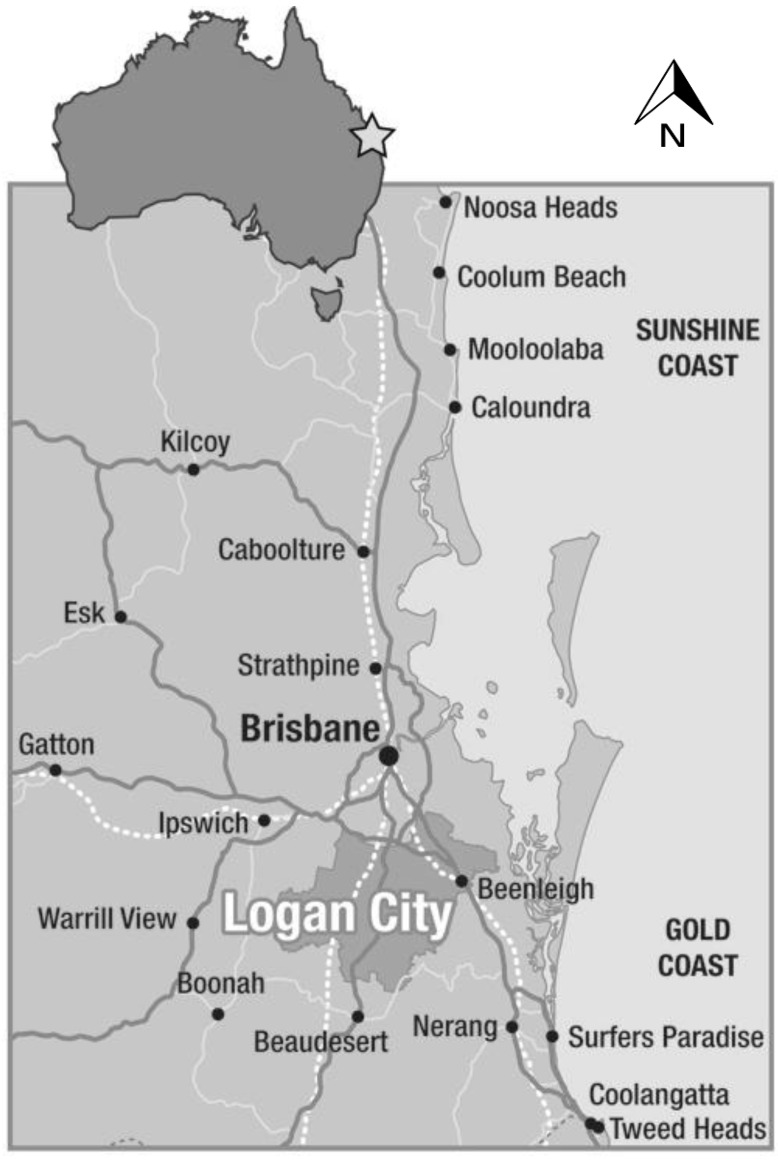
Location of Logan City in South-east Queensland, Australia [35] (Copyright permission was received from the Logan City Council Graphic Designer).

Logan City in south-east Queensland (Figure 1) has an estimated resident population of 300,667 as of 30 June 2013, with 31% of the population residing in the most disadvantaged quintile [33]. It is a growing city that hosts an increasing diversity of cultures. It is a favoured settlement area for migrants due to its close proximity to Brisbane city and the Gold Coast together with the area’s relatively affordable housing and established services and community support networks. Between 2006 and 2012, as a designated refugee settlement zone, almost 900 refugees were resettled in Logan City. Of all local government areas in Australia, Logan is ranked 8th for receiving the most number of humanitarian migrants [34]. There are several well established non-government organisations in Logan that provide a range of settlement, employment and training services to support the social, health and economic needs of humanitarian migrants. It was through links with these organisations that the need for access to land for growing traditional food crops was identified.

**Figure 2:**
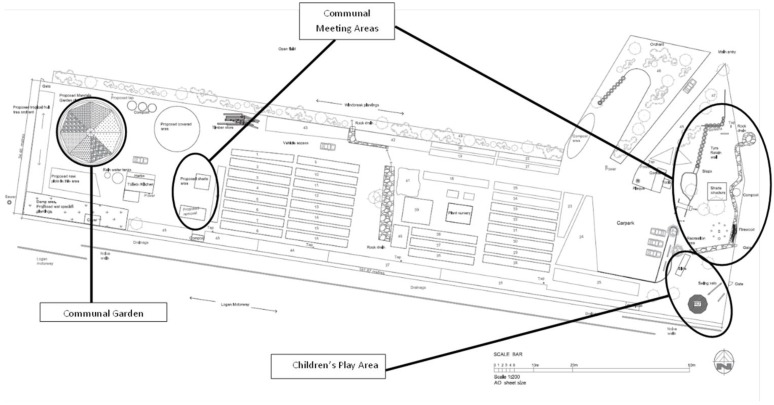
Logan Campus Based Community Food Garden Map.

The Logan campus-based community food garden was established in 2006 with 45 individual plots each approximately 20 m^2^ together with a propagation area, shipping container for equipment and tool storage, several shaded communal areas, basic kitchen facilities and toilets. A map of the garden is presented as Figure 2. The figure shows the 45 individual plots with the important communal areas identified. The garden site is relatively flat with very poor soil quality. There is unlimited access to water from an adjacent lake through a dedicated water pump with underground pipes to taps positioned at strategic locations in the garden. Basic gardening resources are provided free of charge to the gardeners including wheel barrows, shovels, hoes, whipper snipper and lawn mower and soil conditioning products (e.g., gypsum, mulch). These resources have been funded through small grants and funds made available through the university, government departments and community organisations.

The garden has nearly 100 African humanitarian migrants from countries including Congo, Burundi, Somalia and Sudan. All are of lower socio-economic status with most being unemployed and undertaking settlement related classes for training in English language, life and employment skills. Consistent with the profile of the African humanitarian migrants to Australia, most of the gardeners are women with large families of up to seven or eight children. The gardeners work on their individual plots using their own and provided resources to prepare the plots then plant, tend and harvest crops including traditional foods such as cassava and maize and local foods such as pumpkins, bananas, tomatoes and strawberries. In the growing season, gardeners will visit the plot up to four or five times a week to water and tend their crops. As part of the community garden, gardeners, family, friends and others interested in food gardening and traditional foods are involved in the communal spaces for garden based events such as barbeques, meetings and information sessions around food gardening and nutrition.

Members of the local African refugee communities participated in the building of the garden with those interested being assigned plots. There is a waiting list for plots. This food garden has the highest refugee involvement of any community garden in Queensland, with 35 families working individual plots. It has been recognised nationally by the Refugee Council of Australia as one of 29 initiatives demonstrating best practice in settlement, self-sufficiency and participation in the community of newly-arrived humanitarian entrants. It has also been identified as an “important potential gathering place and opportunity for intercultural activity” in the 2006 Logan City Case Study Report developed for the international Intercultural City project.

### 2.2. Data Collection and Analysis

For the present study, semi-structured interviews were conducted, guided by an interview protocol comprising seed questions around participation in the garden. Semi structured interviews are a prominent and widely used data collection strategy in qualitative research and well suited to the purpose and nature of the research being undertaken [36,37]. Interviews were conducted with 12 purposively sampled individual gardeners who were recently settled African humanitarian migrants. Interviews were conducted as a conversation in the Garden setting as a familiar and comfortable location for the participants, recorded and transcribed verbatim. Questions were open ended, short and used easy to understand English to maximise accessibility and to encourage participants to share their views and experience of the garden. Interviews commenced with informed consent, included a couple of warm up questions about the participant’s garden plot and then progressed through three seed questions:

1. Why did you join the garden?
2. Why do you come to the garden?
3. Why is being part of the garden important for you?

Thematic analysis of the transcripts first involved deconstructing participant responses by identifying and grouping key words or phrases throughout the dataset. These groupings together with a copy of the raw data were then shared with a second researcher to conduct a second round of analysis. Once completed, the two coders discussed the findings, clustered the data and named the three themes [38].

Ethics approval for the research was granted through a University Human Research Ethics Committee.

## 3. Results and Discussion

The thematic analysis of the data from the participant interviews revealed three themes: *Land Tenure*; *Re-connecting with Agri-Culture*; and *Community Belonging*.

### 3.1. Theme 1: Land Tenure

The theme *Land Tenure* relates to the refugees having secure access to land (albeit small in size with individual plots being approximately 20 m^2^) they can legitimately farm. The following comments are indicative of this concept:

In Africa it is the citizens who have gardens. The foreigners do not have gardens. Now I have a garden, I feel like a citizen.

(Gardener F)

This quote identifies the perceived link between plot tenure and participation in food gardening with becoming a citizen. It highlights the symbolism and connection with land and place that the garden provides for the migrants. At a more individual level, the next two quotes identify how food gardening is central to the gardeners’ identity and sense of self:

When she digs her own farm and grows the way she knows, then she will eat what she knows.

(Gardener B).

While this quote is more abstract, it relates to using knowledge and skills from the gardener’s past to produce crops that are familiar and of comfort. The next quote identifies the link between where the migrant has come from and their new beginnings facilitated by the secure access to land, in particular the opportunity to plant and grow crops, that the garden has offered:

He is relieved to have a land here to dig. He believes it was very important for an African person, if they didn’t go to school, which majority didn’t, they had to be a farmer. So the closeness was very good, the bond between the human and the soil was of great importance. … This is the only place here for him to dig so that is why it is important for him.

(Gardener D).

Newly arrived humanitarian migrants are invariably housed in rental properties and this means they have no access to land to grow crops and connect with the Earth. Accordingly, access to a physical space that provides the place within which to engage in familiar activities becomes a significant milestone in their transition to their new country of residence [39,40]. Hence, the reference in the first quote to being a citizen. This theme is supported by previous research that has identified the role gardening plays in providing security and developing and maintaining a sense of self and belonging through biographical continuity across time and place between past and present experiences [41].

### 3.2. Theme 2: Re-Connecting with Agri-Culture

The theme *Re-connecting with Agri-Culture* recognises that many African humanitarian migrants have their origins in farming communities or have been involved in small scale food gardens to supplement food available either in their home country or refugee camps [42]. It is part of their being or culture and this is represented in the highlighting of this link by joining agriculture with culture as agri-culture. For these migrants, participation in a community food garden represents the familiar and purposeful activity of growing and harvesting crops, as suggested by the following comments by gardeners:

Both my parents were farmers. It’s knowledge and skills passed down through the generations. I have just been given a plot and am doing everything according to my knowledge and talent basically. I plant them the way I know.

(Gardener B)

This quote shows how participating in the garden offers a place for the migrant to practice their skills and knowledge—to re-connect with and celebrate their family and community background. The following quote shows how growing and harvesting crops offers the opportunity for achievement and feelings of happiness and success:

Remember when the maize grew, that was huge. That was amazing, it was like we had all this stuff.

(Gardener G)

Together these two quotes identify how providing opportunity to apply knowledge and skills from the past can offer a means for migrants to feel more comfortable and capable in their new country of residence. Morgan and Ziglio [43] have described an assets based model of facilitating refugee settlement that views refugees as resourceful people who, with some assistance to adapt to the host culture, are able to address their own issues to achieve wellbeing and self-reliance. One gardener commented:

If you’re a farmer, it covers the whole part of your life, in terms of exercising and in terms of your whole wellbeing. It is very important for me to be in the garden for my whole total wellbeing.

(Gardener E)

This powerful quote summarises how being in the community food garden is important to the “whole total wellbeing” of the gardeners. As such, being in the garden provides migrants the place to practice the skills and life experiences they bring from their country of origin that can be harnessed to assist adaptation to their new context. The freedom to express one’s cultural identity is integral to adapting to new surroundings. Community food gardens offer a place where the skills and life experiences of this population can be practiced and valued [44]. As such, the theme of *Re-connecting with Agri-Culture* specifically recognises the potential of community gardening to connect the migrant’s life experience of agriculture with culture and their transitioning to life in a new country. This finding is consistent with research on community gardens that has demonstrated how participation in a garden offers individuals opportunity to practice and share their knowledge and skills with like-minded others [45].

### 3.3. Theme 3: Community Belonging

The theme *Community Belonging* relates to the role community gardens can play in building relationships and facilitating integration into the society. There is a variety of contexts for the concept of connectedness, though the use of the term in relation to group-level phenomena, such as a sense of caring/cohesion in community settings seems to predominate [46]. Connectedness is associated with positive behavioural, social and mental health outcomes [47]. A sense of belonging, including trust and mutual reciprocity (*i.e.*, social capital [48]), on an individual level, promotes psychological well-being and at a broader level, promotes the social well-being of communities [48,49,50]. Participants consistently suggested involvement in the garden provided opportunity to build connections with others both within and beyond the garden through common interests in produce and farming.

She says when the produce are good it makes her happy because everything is good and she can share with other people. She relates better with people when she has good products and good produce. She feels good about it.

(Gardener B)

*Like last year, I grow maize and peanut in the garden and cassava, so I give it to my friends. It’s a good thing to give it because if I have something they need I can give and if they have something I need, they can give to me also*.

(Gardener A)

These two quotes orient on how participation in the garden provides the individual gardener with produce they can share with others including family, friends and community. This is significant for the gardeners as often traditional crops such as maize can be expensive or difficult to source. As such, the sharing of produce with other community members beyond the garden offers the refugees a way to build their personal status within their local community and with other communities. This is significant in building their social connections and social capital. At a broader level, the following quotes show how participation in the community garden offers gardeners a sense of belonging to a community of life-minded individuals. These quotes identify the building of mutual reciprocity among the gardeners as they must work together and rely on each other to achieve their garden ambitions:

It brings a lot of, not only satisfaction but relief, sense of belonging and you feel people understand you, you feel people… you’re part of that community that you’re in.

(Gardener A)

In the garden there, we work together, yeah .Get things done. Is good because we need to know each other and work together or things won’t finish.

(Gardener D)

The above comments highlight the gardeners’ greater sense of belonging and connectedness through the sharing of produce with other community members, and through working collaboratively with other gardeners. As such, participation in food gardens facilitates processes of inclusion to overcome cultural, social and economic barriers commonly experienced by humanitarian migrants [51]. Much of the research on community gardens has recognised the capacity of gardens to build community and sense of belonging among diverse and often disparate groups through providing safe spaces for shared interests in gardening and associated matters such as land care, sustainable food systems and sustainability [27].

### 3.4. Broader Significance of Findings

The potential for production of culturally appropriate foods in this and other community gardens should not be overlooked, since it has important implications for food security. Access to food is an important determinant of diet and subsequent health outcomes [52,53,54,55,56]. Marginalised and disadvantaged communities and individuals, including refugees [14,57], often reside in so called “food deserts” [58] (*i.e.*, residential areas with poor access to healthy food due to geography, in-store choice or affordability [59,60]). Although the designation of “food desert” often reflects poor access to food *per se*, rather than culturally appropriate food, dietary acculturation and its consequences are likely to be exacerbated by compromised access to culturally appropriate foods [57,14,61,62]. Community gardens are well documented for their ability to positively affect food choice by enhancing knowledge and self-efficacy in relation to production and preparation of vegetables and fruits [21,22,28,63,64]. The strong element of connectedness associated with gardening participation observed in this study implies a pathway for enhanced engagement with community gardens and other community-based health promotion settings. Community gardening presents an opportunity to enhance access to culturally appropriate food, and thus influence food choice [65,66].

Wills and colleagues [67] have noted that community gardens may be more about community than gardening. The perceived benefits of community gardening reported in this study concur with this and revealed a strong desire for refugees to connect to their new country. A qualitative analysis of community garden participants by Kingsley and Townsend [68] showed that community gardens generated a perception of social connectedness amongst urban community members. Thus, although a primary aim of community gardening is to enhance food security, the present study showed that participation may also have flow-on effects on perceived connectedness for refugees.

Community-based public health intervention programs are continually challenged to engage difficult to reach at-risk populations. This single case study suggests that community gardening participation by African refugee families supports social connectedness. Given the single case design of the present study, further research perhaps in the guise of additional case studies of food gardens or other interventions engaging African refugees is needed to increase confidence in the generalizability of the study’s findings. Once understood, these factors have potential to enhance engagement by this vulnerable group in other types of health promotion programs.

## 4. Conclusions

In summary, the concept validity of community gardens as culturally and socially relevant place-based interventions for vulnerable African refugee groups is supported by the reflections of garden participants in the present study. Specifically, community garden participation generates connectedness with their new country through the allocation of tenure of physical space for garden participants; by supporting a reconnection with the purposeful and familiar activity of growing food crops and by promoting a sense of belonging. Integrated within this is the contribution to food security through enhanced access to culturally appropriate foods in an environment that values and builds upon their social and cultural assets. As such, for African humanitarian migrants, participation in a community food garden offers a tangible means to build community connections and connect with their new country.
